# Pilomatrix Carcinoma: Report of Two Cases of the Head and Review of the Literature

**DOI:** 10.3390/curroncol30020109

**Published:** 2023-01-19

**Authors:** Ludovica Toffoli, Giulia Bazzacco, Claudio Conforti, Claudio Guarneri, Roberta Giuffrida, Enrico Zelin, Nicola di Meo, Iris Zalaudek

**Affiliations:** 1 Dermatology Clinic of Trieste, Maggiore Hospital, University of Trieste, 34100 Trieste, Italy; 2 Department of Biomedical and Dental Sciences and Morpho Functional Imaging, University of Messina, 98124 Messina, Italy; 3 Department of Clinical and Experimental Medicine, Dermatology, University of Messina, 98124 Messina, Italy

**Keywords:** pilomatrix, carcinoma, dermoscopy, histopathology, therapy

## Abstract

Background: Pilomatrix carcinoma (PC) is a rare skin tumor arising from hair follicle matrix cells. It is locally aggressive with a high rate of local recurrence after surgical excision. Few cases in the literature have been described and the management is not well defined. Objectives: The aim of this study was to present two cases of PC located on the head and review the relevant literature about epidemiology, clinical and dermoscopic evaluation, characteristics of local and distant metastases, local recurrence rate and management of this rare skin tumor. Methods: We consulted databases from PubMed, Research Gate and Google Scholar, from January 2012 to November 2022. We reviewed the literature and reported two additional cases. Results: We selected 52 tumors in middle-aged to older patients located mostly on the head. Dermoscopy evaluation was rarely performed in the pre-operative diagnostic setting. The most definitive treatment was wide local excision, but local recurrences were common. In total, we observed 11 cases of recurrences and 9 patients with locoregional or distant metastases. Four patients received adjuvant radiotherapy, two patients needed chemotherapy and local cancer therapy and one patient received radiochemotherapy. Conclusion: Our reports and the review of the literature can provide a better awareness and management of this rare tumor.

## 1. Introduction

Pilomatrix carcinoma (PC) is a rare skin tumor arising from hair follicle matrix cells and it is most frequently located on the head and neck region of male patients (fifth to seventh decades) [1]. PC corresponds to the malignant variant of pilomatricoma and is locally aggressive with a high rate of local recurrence after surgical excision [1]. The exact metastatic potential is not clear, but metastases and mortality have been described in the literature [2,3,4,5,6].

PC usually presents as a rapidly growing nodular lesion and dermoscopy may be helpful for improving its recognition, even if the diagnosis of malignancy is based on histological examination.

Few cases in the literature have been described and the management is not well defined. To better describe the epidemiologic characteristics, clinical features, therapeutic options and follow-up of this rare tumor, we reviewed the literature and reported two additional cases.

The aim of this study was to present two cases of PC located on the head and to review the literature evaluating epidemiology (age, sex, distribution), clinical and dermoscopic evaluation, characteristics of local and distant metastases, local recurrence rate and management (follow-up and treatment) of this rare tumor.

## 2. Methods

We found the relevant literature by searching different databases: PubMed, Research Gate and Google Scholar. We used the following combination of key words: “pilomatrix carcinoma”, “pilomatrical carcinoma”, “malignant pilomatricoma”, from January 2012 to November 2022. In total, 48 papers were identified. Inclusion criteria were systematic review or meta-analysis of randomized controlled trials, review, retrospective comparative reviews/studies and case series. Exclusion criteria were laboratory studies and non-English translated articles. A wide review of the bibliography of each of the selected articles was performed. In total, 37 papers met our inclusion criteria, including 1 review [7] and 36 case reports and case series [2,3,4,5,6,8,9,10,11,12,13,14,15,16,17,18,19,20,21,22,23,24,25,26,27,28,29,30,31,32,33,34,35,36,37,38] (Figure S1).

## 3. Results

### 3.1. Case Reports

We present two cases of a PC on the head.

**Figure 1:**
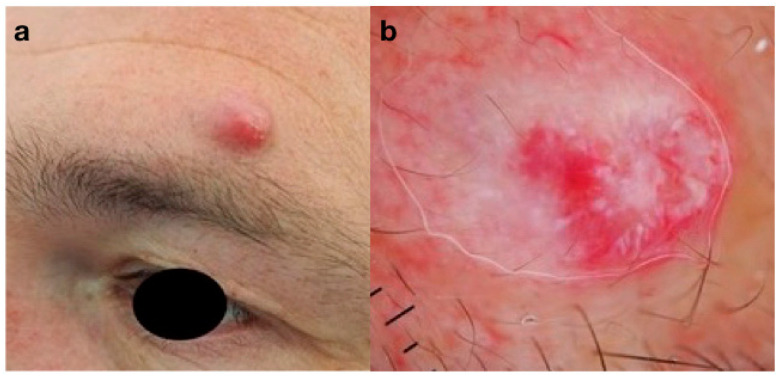
(**a**) Clinical aspect of a pilomatrix carcinoma (PC): asymptomatic pinkish-red nodule of the left supraorbital area; (**b**) dermoscopy of PC: homogeneous structureless pinkish/red background, white/yellowish blotches, predominant arborizing vascular pattern.

The first patient, a 54-year-old man, arrived for consultation in our department for a fast-growing asymptomatic nodule localized in the left supraorbital area. Clinical examination revealed a solitary, reddish, firm raised nodule, 12 mm in diameter without locoregional lymphadenopathy (Figure 1a). Dermoscopic evaluation showed a homogeneous structureless pinkish/red background with some white/yellowish blotches and a predominant arborizing vascular pattern (Figure 1b). A surgical excision of the lesion was planned and the histopathology examination revealed a PC with nests of atypical basaloid cells, high presence of ghost cells, increased mitotic activity and necrosis. An ultrasound was performed to exclude lymphatic localizations in the head and neck area. A wide, local excision of the tumor with 5 mm peripheral margins was performed, but the histopathological report showed the presence of pilomatrical tumor with ghost-cells in the deepest border. A further surgical re-excision (5 mm excision margins) was performed, obtaining clear margins. The patient is now followed every 6 months without signs of tumor relapse.

**Figure 2:**
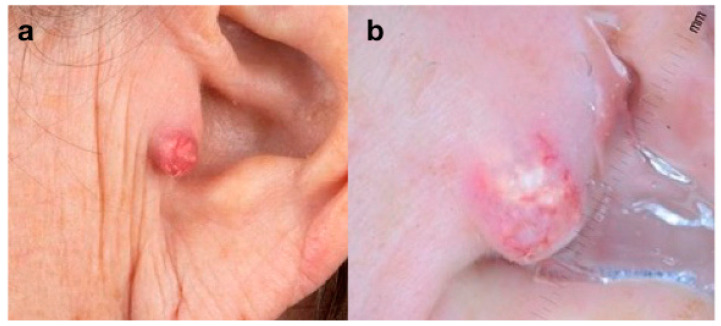
(**a**) Clinical aspect of a pilomatrix carcinoma (PC): asymptomatic erythematous firm nodule of the left preauricular area; (**b**) dermoscopy of PC: pinkish/red background with white/yellowish structureless areas surmounted by focused hairpin and branched vessels.

The second case concerns an otherwise healthy 65-year-old women with an asymptomatic erythematous, firm, 14 mm in diameter nodule located on the left preauricular area (Figure 2a). Dermoscopy revealed a pinkish/red background with white yellowish structureless areas surmounted by focused hairpin and branched vessels (Figure 2b). The lesion was removed with clear margins and the histopathology report showed findings consistent with PC. An ultrasound was performed with no evidence of lymphadenopathy in the head and neck area. The patient is scheduled for biannual skin checks. At the 12-month follow-up visit, no signs of tumor recurrence were detected.

### 3.2. Review

#### 3.2.1. Age and Sex

In the last 10 years (from 2012 to 2022), 52 cases of PC have been described in the literature, making this tumor very rare. There was a male predominance with 29 male patients (55.8%) compared to 23 female patients (44.2%) (male to female ratio 1.3:1). Age at presentation ranged from 8 months to 87 years (mean 57 years, median 63 years). Lesions occurred most in the sixth and seventh decades (52%) (Table 1).

#### 3.2.2. Distribution

All the studies reported the location of the lesions. PCs were mostly located on the head (*N* = 32/52, 62%), followed by the upper extremity (*N* = 6/52, 11%) trunk (*N* = 6/52, 11%), lower extremity (*N* = 4/52, 8%), neck (*N* = 3/52, 6%) and genitalia (*N* = 1/52, 2%). No patient had multiple lesions (Table 1).

#### 3.2.3. Clinical Presentation and Lesion Size

Information regarding initial clinical presentation was available for all patients. The lesion was most frequently described as an asymptomatic, firm, non-tender nodule with frequent rapidly growing behavior. None of the lesions described with accuracy were correctly diagnosed before biopsy and histological evaluation.

Size of the PC was available for 46 lesions (89%). Tumor diameters ranged from 0.5 to 15 cm (mean 2.5 cm, median 2 cm). Rapid growth, either of new lesion or stable lesion from several months was the most frequent motivation for biopsy and diagnosis (Table 1).

#### 3.2.4. Metastases

Locoregional or distant metastases occurred in nine patients (17%) [2,4,5,6,7,16,18,22,34]. Two patients showed metastases at first tumor staging after primary tumor resection. They both presented multiple lung metastases and pathological thoracic lymph nodes at the CT scan [4,34].

New findings showed that metastases occurred in six patients with previously treated PC [2,5,6,16,18,22]. Of these findings, at three months following wide local excision of a neck PC, the first patient complained of persistent posterior neck pain secondary to the cervical vertebral bone extension [6]. The second patient, eight months after diagnosis of a scalp PC, presented three local recurrences with bone infiltration and lung metastasis [16]. Similarly, another patient presented local recurrence and parotid gland metastases 12 months after local excision of preauricular PC [2]. The fourth case was a patient with altered sensorium and increased intracranial pressure due to an invasion of the skull bones and brain in the cavernous sinus and draining veins 16 months after a wide excision of PC of the scalp [5]. The fifth patient, six months after the excision of a forehead PC, demonstrated metastases at the cervical intraparotid lymph nodes [18]. Another case, 10 months after excision of a scalp PC, showed diffuse metastases on lung, pleura, liver and bones at the PET-CT scan [22]. The last patient at a six-month follow-up for a wide local excision of a PC of the cheek showed lymph node metastases [7]. A summary of the results of locoregional or distant metastases is reported in Table 2.

#### 3.2.5. Dermoscopy

Dermoscopic examination was reported in only three patients [23,27,32]. The first case was a 3 cm smooth pink to violaceous nodule in the forehead region which revealed focal ulceration and irregularly shaped telangiectasias at dermoscopy [23]. The second one was a reddish–violaceous, ulcerated nodule (3 cm in size) on the mandibular region which revealed telangiectasias, white structureless areas, yellowish hues and erythematous background at dermoscopy [27]. The third case showed a reddish nodule 1.4 cm in diameter with asymmetrically distributing white/yellowish blotches, homogeneous structureless purple/blue areas and predominant arborizing vessels [32].

#### 3.2.6. Method of Treatment and Recurrence

All 52 patients had a histological diagnosis of PC. A first incisional biopsy was performed in five lesions of the head and upper extremities (10%), followed by a wide local excision. Only one patient died before re-excision [4].

In total, a wide local excision was reported for 32 lesions (62%). Mohs micrographic surgery (MMS) was chosen for four tumors [19,20,21,23]; two of these achieved histologic clearance with 5 mm margin MMS. None of these cases had recurrences or metastasis.

Eckhoff et al. excised the lesion and left the skin defect uncovered pending final pathology, then they re-excised after 12 days to obtain 5 mm margins and subsequently cover the skin with a graft [24]. The patient showed a recurrence after 4 months from surgery, but the initial dimension was very large (15 × 12 cm) with uncontrolled hemorrhage.

In total, 13 PCs were simply excised, without reported margins of excision and one case was first excised and then re-excised to obtain 2 mm safety margin after diagnosis [26]. In two cases, multiple excisions were reported in order to achieve clear margins [14,18].

Due to lymph node infiltration, one patient experienced a neck dissection [2]; instead, two patients with associated regional morphologically abnormal lymph nodes underwent lymph node dissection, but the removed specimen was negative for malignancy [3,13]. Radiotherapy (RT) was performed in one patient following excision of the primary tumor [13], in two patients after diagnosis of recurrences [2,29] and in one case after lymph nodes metastasis [7]. After surgery of the primary tumor, radiation therapy was recommended in the first case with a close margin [13], another patient received fractionated external beam radiation therapy to the left parotid region and neck [2] and the patient with lymph nodes metastasis received adjuvant radiation therapy [7]. In one case, adjuvant RT was used for local recurrence [29].

Two patients needed chemotherapy (CT) and local cancer therapy [26,34] and one patient received radiochemotherapy [16]. Systemic cancer therapy was given in case of metastasis; the patient with PC of the scalp and lung metastases achieved a complete response with oral cyclophosphamide and etoposide [34]. Bevayeli et al. described a case of PC located in the caruncle of the right eye completely excised with a 2 mm safety margin, bevacizumab as eye drops was added four times per day for three months [26].

Radiochemotherapy was performed in case of extensive local invasion and metastasis: first line CT therapy with 5-Fluorouracil and cisplatin, second line with gemcitabine and taxotere, third line with irinotecan and bevacizumab, fourth line with cyclophosphamide and vinblastine [16]. A summary of the treatments is reported in Table 3.

In total 11 cases of recurrences were observed (*N* = 11/52, 21%), at an average of 6 months after surgery (range 2 to 16 months) [2,5,6,7,15,16,22,24,29,31]. After the first wide local excision, three cases recurred (*N* = 6/52, 6%), one case 2 months after surgery (the other one not reported) and 6 lesions simply excised recurred at an average of 6 months (*N* = 6/52,12%). One patient, with malignant PC with involvement of the base and margins of the specimen, refused radiotherapy post-surgery and presented recurrence and fatal metastasis after 16 months from diagnosis [5]. In total, two patients showed multiple episodes of recurrence [2,16].

#### 3.2.7. Follow-Up

Follow-up information was not reported in six cases (*N* = 6/52, 12%) [11,12,14,25,32,35]. The majority of patients (*N* = 41/52, 79%) had a regular follow-up (range from one month to eight years), receiving biannual dermatologic visits and in some cases also had a radiological examination. The follow-up imaging procedures used were chest radiography [5], ultrasound [18,31], CT scan [19], 18F-FDG PET/CT scan [22], alternating MRI and PET-CT scans [28], and radiological imaging was not specified [2,27].

One patient was lost to follow-up [13]. Four patients died; three of these patients presented metastases and one expired due to liver failure secondary to congestive heart failure [3,4,5,6].

Characteristics of the 52 cases of PC in the recent literature (2012–2022) are reported in Table 4.

## 4. Discussion

In the literature, benign pilomatricoma shows a female predilection and occurs usually in children and young adults with a small peak of onset in the elderly [39], whereas PC commonly arises later in adults or the elderly with a male predominance. This review of the literature of the last 10 years confirmed that PC has a slight male predominance (male to female ratio around 1.3:1) and occurs mostly in the sixth and seventh decades.

Even if multiple pilomatricomas have been associated with some particular disease including Turner syndrome, Gardner syndrome, myotonic dystrophy, Steinert disease and sarcoidosis [39], we did not find any syndrome or genetic condition associated with PC, as in the review of Jones 2017 et al. [1].

Clinically, PC shares some features with pilomatricoma, in fact it is a firm asymptomatic nodule typically found in the head and neck area, but unlike the benign variant, PC frequently shows a rapid growth [1,7]. In previous papers, the prevalent sites of PC were the head and neck region, followed by upper extremities, trunk and lower extremities [1,14]. Our results partly reflected these findings; we noted that head was the most frequent localization, followed by upper extremity, trunk, lower extremity, neck and genitalia. Moreover, all the tumors were solitary lesions with a diameter of around 2 cm.

Dermoscopy evaluation was rarely performed/reported in pre-operative diagnostic settings although it is a useful tool in the context of a rare and difficult to suspect tumor. The most frequent dermoscopic characteristics were purple/blue background with white/yellow blotches, irregularly-shaped telangiectasias, arborized vessels and focal ulceration. White/yellowish blotches can also be found in pilomatricoma because of the presence of calcifications and cornified substance, instead ulceration is rarely found in pilomatricoma and the arborizing vessels have never been observed in this benign tumor [32]. Unlike the vascular pattern of nodular basal cell carcinoma, vessels in PC are subtler, numerous and out of focus [32].

Wide local excision was the most frequent therapeutic option due to the relative high recurrence rate reported in the literature [7]. Radical excision with histologically confirmed that negative margins are usually recommended; however, the peritumoral margins varied considerably (from 5 mm to 2 cm). Considering the tumor recurrence after surgery, MMS was successfully completed in four cases, allowing precise margin control. As already assessed in the review of Jones 2017 et al., this method potentially can improve patient outcomes [1]. The invasive infiltration of PC is significant and relapses are frequent (21%), even after wide local excision. Simple excision showed a higher recurrence rate than wide local excision or MMS [1].

In previous studies, distant metastases were uncommon and occurred in 10–13% of cases [1,7,40]. In the present review, we observed nine patients (17%) with metastatic disease; of these, two (4%) showed metastases at the first tumor staging after primary tumor resection, while six (12%) showed metastases during the follow-up. The prevalent sites were lung and regional lymph nodes, as reported in the literature [1], followed by local bone infiltration, skull bones, brain and parotid gland. Only one case showed diffuse metastases on the lung, pleura, liver and bones.

Furthermore, we observed three deaths resulting directly or indirectly from PC invasion.

Adjuvant RT was used to treat local recurrence and metastatic disease and it was also recommended due to one close margin, after multidisciplinary discussion.

Although some authors considered CT and RT to be not effective in disease control [40,41,42], in our review, the disease showed a response to these treatments. Systemic CT was given for PC of the scalp and lung metastases, achieving a complete response with oral cyclophosphamide and etoposide [34]. Interestingly, a topical therapy with bevacizumab was also applied in the case of the caruncle of the eye in order to prevent recurrence after surgery [26]. This is the first reported case of PC of the ocular surface and there is no standard topical chemotherapy protocol. Bevacizumab is a VEGF-targeting monoclonal antibody with an anti-angiogenetic role indicated for the treatment of many cancers [43]. Even if the topical formulation of this drug is not approved for this type of tumor, it is considered a good and safe option because systemic bevacizumab was already used for advanced PC [26] and topical bevacizumab has shown efficacy in ocular surface squamous neoplasia [44,45]. Radiochemotherapy was performed in the case of an extensive local infiltration and metastases with adequate control of the disease (4-year follow-up) [16].

Follow-up information was not reported in all cases, but the majority of patients received regular clinical examination. There are no well-defined recommendations for follow-up; however, the patients should be closely monitored with ultrasound and other imaging examinations if necessary. The role and the type of follow-up imaging are still unclear, it depends on the patient history and the clinical examination.

## 5. Conclusions

PC is a rare skin tumor arising from hair follicle matrix cells. It is a lesion with non-specific features and dermoscopy evaluation can be helpful for improving the clinical suspicion. The excision of doubtful nodular lesions is mandatory especially in adults, because PC carcinoma has not only a strong tendency to recur locally, but it can also metastasize. However due to its rarity, there are no well-defined guidelines for the management. A complete surgical excision with clear margins is the best therapeutic option and a regular follow-up is recommended overall.

These two case reports and the review of the literature can give a better awareness and management of this rare tumor.

## Figures and Tables

**Table 1:** Patient demographics and characteristics of pilomatrix carcinoma.

**Sex (*n*, %)**	
Male	29 (55.8%)
Female	23 (44.2%)
**Mean age (years, range)**	57 (8 months-87)
**Distribution (*n*, %)**	
Head	32 (62%)
Trunk	6 (11%)
Upper extremity	6 (11%)
Lower extremity	4 (8%)
Neck	3 (6%)
Genitalia	1 (2%)
* **Mean size (cm, range)**	2.5 (0.5–15)

* Data available for 46 lesions (89%).

**Table 2:** Summary of cases with locoregional or distant metastases.

Location of Metastasis	References	No. of Patients	Location of PC	Time of Onset (months)
Lung, lymph nodes	Vadrucci et al. [4] Arslan et al. [34]	2	Head	At time of diagnosis
Bone	Walker et al. [6]	1	Neck	3
Lung, bone	Sorin et al. [16]	1	Head	8
Parotid gland	Liu et al. [2]	1	Head	12
Brain, bone	Flynn et al. [5]	1	Head	16
Lymph nodes	Otero et al. [18]	1	Head	6
Lung-pleura, liver, bone	Sengoz et al. [22]	1	Head	10
Lymph nodes	Errmann et al. [7]	1	Head	6

Abbreviation: PC = pilomatrix carcinoma.

**Table 3:** Summary of treatments.

Treatment ***N*** = 52	
**Surgery**	
Wide local excision	30
Simple/incisional excision	15
MMS [19,20,21,23]	4
Excision + LND [2,3,13]	3
**Adjuvant therapy (after surgery)**	
RT [2,7,13,29]	4
CT [34]	1
CT + RT [16]	1
Bevacizumab (eye drops) [26]	1

Abbreviations: MMS = Mohs micrographic surgery, CT = chemotherapy, RT = radiotherapy, LND = lymph node dissection.

**Table 4:** Characteristics of 52 cases of pilomatrix carcinoma of the last 10 years literature (2012–2022). Number of patients, age (years), distribution (head, neck, upper or lower extremities, genitalia), clinical presentation, size (cm), dermoscopic description, metastasis and time of onset, follow-up and its duration, therapy with margins of surgical excision (cm), other therapy, local recurrence.

References	Patients (n°)	Age (years)	Gender	Site	Presentation	Size (cm)	Dermoscopy	Metastasis (Time of Onset)	Follow-Up (Duration)	Therapy (Margins)	Other Therapy	Local Recurrence
Karaaslan O et al. [33]	1	65	M	Head	Ulcerated nodule	1.5	NR	-	Periodically (6 months)	Wide local excision (1 cm)	-	-
Arslan D et al. [34]	1	76	M	Head	Asymptomatic nodule	NR	NR	Lymph nodes, lung (at time of diagnosis)	Periodically (6 months)	Excision	CT	-
Tvrdi AB et al. [35]	1	10	F	Head	Firm, asymptomatic nodule	0.9	NR	-	NR	Wide local excision	-	-
Mukherjee B et al. [36]	1	65	F	Head	Asymptomatic nodule	1.2	NR	-	Periodically (NR)	Excision	-	-
Alcántara-González J et al. [37]	1	87	M	Head	Firm, ulcerated nodule	1.5	NR	-	Periodically (12 months)	Incisional biopsy, Wide local excision	Re-excision	-
Sato S et al. [38]	1	36	M	Upper extremity	Ucerated nodule	1	NR	-	Periodically (6 months)	Incisional biopsy, Wide local excision (5 mm)	-	-
Pauli M et al. [8]	1	68	F	Head	Firm, asymptomatic nodule	2	NR	-	Periodically (12 months)	Incisional biopsy, wide local excision (4 mm)		-
Song M et al. [9]	1	30	F	Genitalia	Firm asymptomatic nodule	3	NR	-	Periodically (8 years)	Excision	Wide local excision (1 cm)	-
Walker DM et al. [6]	1	43	F	Neck	Firm mobile symptomatic nodule	3	NR	Vertebral bone (after 3 months)	Exitus	Excision	Metastasis excision, re-excision (CT not initiated)	1 (after 3 months)
Vadrucci M et al. [4]	1	76	M	Head	Soft-tissue nodule	NR	NR	Lymph nodes, lung (at time of diagnosis)	Exitus after 3 months	Incisional biopsy	-	-
Parra L et al. [10]	1	58	M	Lower extremity	Firm, asymptomatic, ulcerated nodule	6.5	NR	-	every 6 months (12 months)	Wide local excision (1 cm)	-	-
Villada G et al. [11]	1	79	F	Lower extremity	Ulcerated, aymptomatic nodule	2.2	NR	-	NR	Excision	-	-
Bailey EE et al. [12]	1	84	M	Upper extremity	Nodule	NR	NR	-	NR	Excision	-	-
Lohiya S et al. [13]	1	60	M	Head	Firm, asymptomatic ulcerated nodule	9	NR	-	Lost	Wide local excision (5 mm) + lymph node dissection	Adiuvant RT (close margin)	-
Cornejo KM et al. [14]	1	44	M	Trunk	Asymptomatic nodule	NR	NR	-	NR	Excision	Wide local excision, re-excision	-
Gupta M et al. [15]	1	25	M	Neck	Firm, mobile, asymptomatic nodule	2	NR	-	Periodically (1 year)	Excision (after FNAC)	Recurrance wide local excision	1 (after 3 months)
Sorin T et al. [16]	1	15	F	Head	Nodule	2	NR	Parietal bone, superior sagittal sinus, lung metastases (after 8 months)	Periodically (4 years)	Excision	Recurrence wide local excision (2 cm), RT, CT	3 (after 5, 3, 4 months)
Alloui M et al. [17]	2	63	F	Lower extremity	Multinodular lesion	5	NR	-	Periodically (2 years)	Incisional biopsy, wide local excision (1 cm)	-	-
		66	F	Head	Nodule	1.5	NR	-	Periodically (NR)	Wide local excision	-	-
Liu JF et al. [2]	1	46	F	Head	Firm, asymptomatic nodule	1	NR	Parotid gland (after 12 months)	Periodically follow-up + imaging (2 years)	Excision	Recurrence excision + lymph node dissection, RT	2 (after 1 year, 3)
Flynn et al. [5]	2	24	F	Head	Lobulated nodule	12	NR	Skull bones, brain (after 16 months)	Exitus	Excision	RT refused (involvement of base and margins)	1 (after 16 months)
		14	F	Upper extremity	Firm, nontender nodule	2	NR	-	Yearly follow-up + chest X-Ray (12 months)	Excision	Wide local excision (ose margin)	-
Otero MN et al. [18]	1	8	F	Head	Asynnetric nodule	9	NR	Lymph nodes (after 6 months)	Periodically follow-up + ultrasound (6 months)	Wide local excision	Re-excision	-
Xing L [19]	2	68	F	Head	Nodule	1.9	NR	-	Periodically (7.5 months)	Mohs surgery (5 mm)	-	-
		68	M	Head	Rapidly growing nodule	1.1	NR	-	Periodically follow-up + CT imaging (6 months)	Mohs surgery (5 mm)	Mohs surgery (depth)	-
Xim JS et al. [20]	1	8-month	M	Head	Nodule	0.7	NR	-	Periodically (12 months)	Excision	Wide local excision (1 cm)	-
Fernandez-Florez A et al. [21]	1	78	M	Upper extramity	Ulcerated plaque	0.6	NR	-	Periodically (8 months)	Mohs surgery	-	-
Martin S et al. [3]	1	74	M	Head	Nodule with associated regional lymphadenopathy	4	NR	-	Exitus	Wide local excision + level II and III neck dissection	-	-
Sengoz T et al. [22]	1	37	F	Head	Nodule	NR	NR	Lung, pleura, liver, bone (after 10 months)	Periodically follow-up with 18F-FDG PET/CT (10 months)	Excision	-	1 (after 10 months)
White C et al. [23]	1	62	M	Head	Nodule	3	Focal ulceration, irregularly telangiectasias	-	6-monthly follow-up (14 months)	Excision	Mohs surgery	-
Eckhoff MD et al. [24]	1	46	M	Upper extremity	Nodule	15	NR	-	Periodically(24 months)	Excision margin-controlled	Recurrence excision	1 (after 4 months)
Yeo MK et al. [25]	1	43	F	Head	Nodule	2.1	NR	-	NR	Excision	no	-
Harbiyeli II et al. [26]	1	45	F	Head	Nontender nodule	1.5	NR	-	Periodically 12 months)	Excision	Re-excision (2 mm safety margin) + bevacizumab eye drops	-
Dell’Antonia M et al. [27]	1	80	M	Head	Rapidly growing, ulcerated nodule	3.5	Telangiectasias, white structureless areas, yellowish hues, erythematous background	-	6-monthly follow-up + imaging (5 years)	Wide local excision	-	-
Subramanyam et al. [28]	1	51	M	Lower extremity	Slow-growing nodule	6	NR	-	3-monthly follow-up for the first 2 years (MRI and PET-CT scans)	Partial excision, wide local excision	-	-
Papadakis M et al. [29]	1	79	F	Head	Slow growing nontender nodule	NR	NR	-	Periodically (4 years)	Wide local excision	Recurrence excision + adjuvant RT	1 (after 2 months)
Briley T et al. [30]	1	51	M	Trunk	Rapidly growing nontender nodule	4	NR	-	1 month	Wide local excision (1 cm)	-	-
Herrmann JL et al. [7]	13	72	M	Neck	Rapidly growing nodule	1	NR	-	Periodically (>6 months)	Wide local excision	-	-
		68	M	Head	Rapidly growing nodule	0.8	NR	-	Periodically (>6 months)	Wide local excision	-	1 (NR)
		67	M	Head	Ulcerated papule	0.8	NR	-	Periodically (>6 months)	Wide local excision	-	-
		78	M	Head	Rapidly growing nodule	2	NR	-	Periodically (>6 months)	Wide local excision	-	-
		62	M	Head	Rapidly growing nodule	1	NR	-	Periodically (>6 months)	Wide local excision	-	-
		67	M	Head	Ulcerated nodule	3	NR	-	Periodically (>6 months)	Wide local excision	-	-
		78	M	Trunk	Rapidly growing nodule	0.8	NR	-	Periodically (>6 months)	Wide local excision	-	-
		76	M	Trunk	Rapidly growing nodule	0.9	NR	-	Periodically (>6 months)	Wide local excision	-	-
		59	F	Head	Rapidly growing nodule	0.5	NR	Lymph nodes (after 6 months)	Periodically (>6 months)	Wide local excision	Adjuvant RT (lymph nodes)	-
		51	F	Head	Rapidly growing nodule	1.5	NR	-	Periodically (>6 months)	Wide local excision	-	1 (NR)
		63	F	Upper extremity	Rapidly growing nodule	2	NR	-	Periodically (>6 months)	Wide local excision	-	-
		71	F	Trunk	Rapidly growing nodule	3	NR	-	Periodically (>6 months)	Wide local excision	-	-
		69	F	Trunk	Rapidly growing nodule	3.5	NR	-	Periodically (>6 months)	Wide local excision	-	-
Weng G et al. [31]	1	53	F	Trunk	Tenacious subcutaneous nodule	2	NR	-	Periocally follow-up + ultrasound (4 years)	Excision	Recurrence excision (RT refused)	1 (after 7 months)
Ravaioli GM [32]	1	69	M	Head	Rapidly growing reddish nodule	1.4	Arborized vessels, purple/blue areas, white/yellowish blotches	-	NR	Excision	-	NR

Abbreviations: M = male, F = female, NR= not reported, CT = chemotherapy, RT= radiotherapy, PET= positron emission tomography, FNAC = fine needle aspiration cytology.

## Data Availability

The data presented in this study are available on request from the corresponding author.

## Supplementary Materials

The following supporting information can be downloaded at: https://www.mdpi.com/article/10.3390/curroncol30020109/s1, Figure S1: PRISMA 2020 flow diagram for new systematic reviews which included searches of databases and registers only. Ref. [46] cited in Supplementary Materials.
