# Supplementary material for: Pilomatrix Carcinoma: Report of Two Cases of the Head and Review of the Literature
Source: Curr Oncol. 2023 Jan 19;30(2):1426–38. doi: 10.3390/curroncol30020109 (PMC9955132; doi:10.3390/curroncol30020109)
Supplement: Supplementary file 1 [file curroncol-30-00109-s001.zip › curroncol-2117207-supplementary.pdf]

Current Oncology

MDPI

Supplementals

Pilomatrix Carcinoma: Report of Two Cases of the Head and Review of the Literature

Ludovica Toffoli [1], Giulia Bazzacco [1,*], Claudio Conforti [1], Claudio Guarneri [2], Roberta Giuffrida [3,*], Enrico Zelin [1], Nicola di Meo [1] and Iris Zalaudek [1]

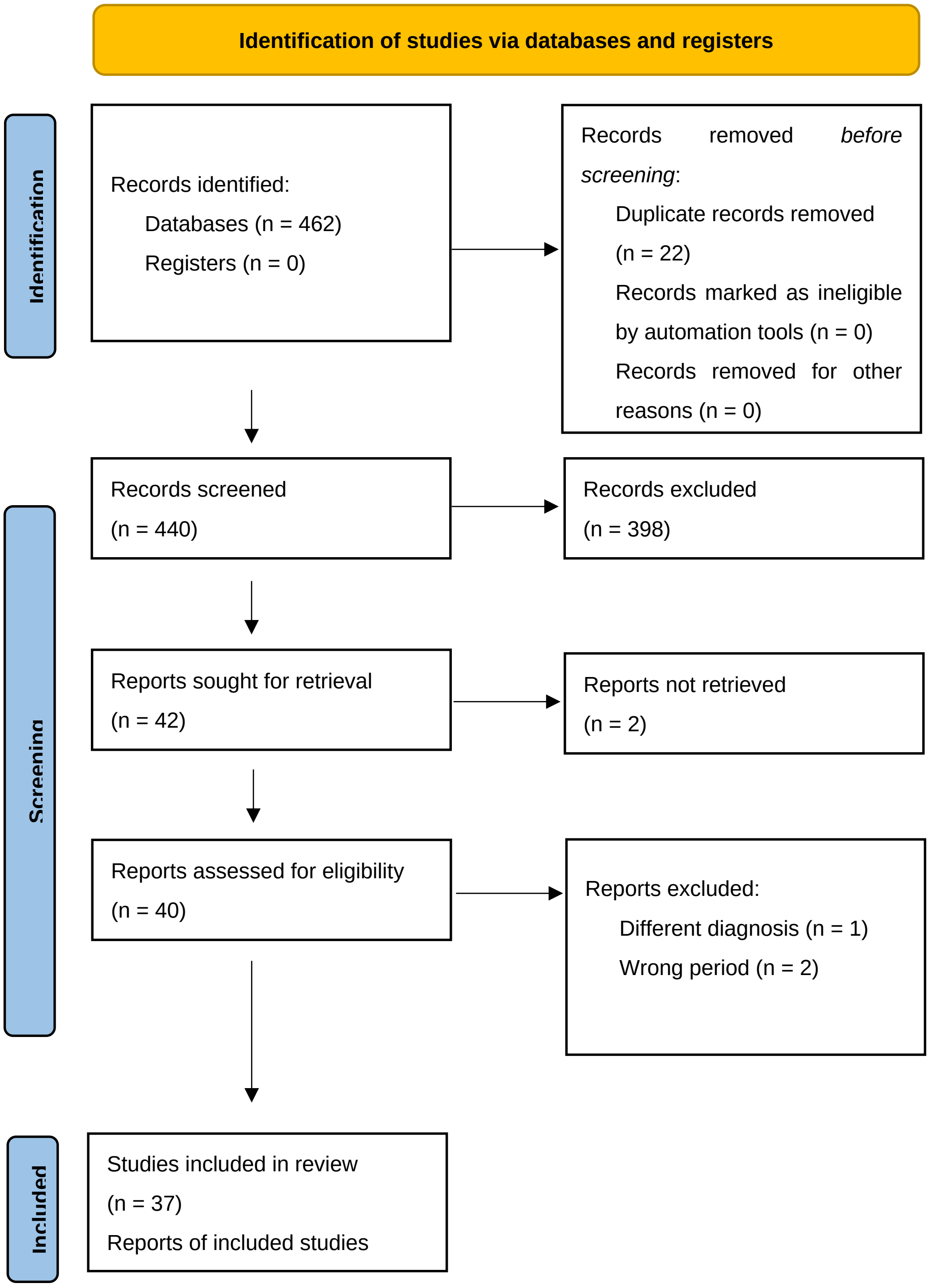

Figure S1. PRISMA 2020 flow diagram for new systematic reviews which included searches of databases and registers only.

From: Page MJ, McKenzie JE, Bossuyt PM, Boutron I, Hoffmann TC, Mulrow CD, et al. The PRISMA 2020 statement: an updated guideline for reporting systematic reviews. BMJ 2021;372:n71. doi: 10.1136/bmj.n71

For more information, visit: http://www.prisma-statement.org/
